# Prismatic Silver Nanoparticles Decorated on Graphene Oxide Sheets for Superior Antibacterial Activity

**DOI:** 10.3390/pharmaceutics14050924

**Published:** 2022-04-24

**Authors:** Thi Tuong Vi Truong, Chien-Chang Chen, Selvaraj Rajesh Kumar, Chih-Chien Hu, Dave W. Chen, Yu-Kuo Liu, Shingjiang Jessie Lue

**Affiliations:** 1 Division of Pediatric Gastroenterology and Hepatology, Department of Pediatrics, Chang Gung Memorial Hospital, Taoyuan 333, Taiwan; truongthituongvi005@gmail.com (T.T.V.T.); cgj2841@cgmh.org.tw (C.-C.C.); 2 Department of Chemical and Materials Engineering, Chang Gung University, Taoyuan 333, Taiwan; rajeshkumarnst@gmail.com (S.R.K.); ykliu@mail.cgu.edu.tw (Y.-K.L.); 3 College of Medicine, Chang Gung University, Taoyuan 333, Taiwan; 4 Department of Orthopedics, Chang Gung Memorial Hospital, Taoyuan 333, Taiwan; chihchienhu@hotmail.com; 5 Department of Orthopedic Surgery, Chang Gung Memorial Hospital, Keelung City 204, Taiwan; mr5181@cgmh.org.tw; 6 Department of Safety, Health and Environment Engineering, Ming-Chi University of Technology, New Taipei City 243, Taiwan

**Keywords:** silver nanoparticles, nanoprisms, nanoparticles, graphene oxide, antibacterial activity

## Abstract

Spherical silver nanoparticles (Ag NPs) and silver nanoprisms (Ag NPrsms) were synthesized and decorated on graphene oxide (GO) nanosheets. The Ag contents were 29% and 23% in the GO–Ag NPs and GO–Ag NPrsms, respectively. The Ag NPrsms exhibited stronger (111) crystal signal than Ag NPs. The GO–Ag NPrsms exhibited higher Ag (I) content (75.6%) than GO-Ag NPs (69.9%). Increasing the nanomaterial concentration from 25 to 100 µg mL^−1^ improved the bactericidal efficiency, and the antibacterial potency was in the order: GO–Ag NPrsms > GO–Ag NPs > Ag NPrsms > Ag NPs > GO. Gram-positive *Staphylococcus aureus* (*S. aureus*) was more vulnerable than Gram-negative *Escherichia coli* (*E. coli*) upon exposure to these nanomaterials. The GO–Ag NPrsms demonstrated a complete (100%) bactericidal effect against *S. aureus* at a concentration of 100 µg mL^−1^. The GO–Ag composites outperformed those of Ag or GO due to the synergistic effect of bacteriostatic Ag particles and GO affinity toward bacteria. The levels of reactive oxygen species produced in the bacteria–nanomaterial mixtures were highly correlated to the antibacterial efficacy values. The GO–Ag NPrsms are promising as bactericidal agents to suppress biofilm formation and inhibit bacterial infection.

## 1. Introduction

Graphene oxide (GO) has recently been a focus of interest due to its two-dimensional, honeycomb-like lattice structure, which contains many functional groups attached to both sides of the carbon planes and on their edges. Functional groups, such as carbonyl (–COOH) and hydroxyl (–OH), functionalize the nanoparticles, enhancing the reactivity of GO. This is of use in various applications, including energy, electronics, and environmental and biomedical applications [1].

As regards biological function, GO has versatile characteristics and can be used as a disinfectant material or drug delivery vehicle because of its biocompatibility. Furthermore, it is effective at inhibiting the growth of harmful micro-organisms [2]. Its bactericidal mechanism is currently understood in terms of its physical and chemical functions. For instance, GO can destroy the bacterial outer membrane structure by interacting with the phospholipid layer on the surface, and it can penetrate perpendicularly into the inner bacterial cytoplasm, destroying the morphological structure of the bacteria [3].

Metallic-based nanoparticles, such as silver, copper, and iron, exhibit unique characteristics because of their high surface-area-to-volume ratios, which result in distinct chemical, mechanical, and electrical properties different from those of the bulk metals [4,5,6]. In particular, silver (Ag) nanoparticles are among the most effective metals to possess bactericidal functionality [7]. Several mechanisms were proposed to explain such antibacterial properties. For instance, the Ag ion can penetrate into the cytoplasm of the bacterial membrane, causing oxygen stress throughout the membrane, thus destroying the bacterial structure and integral morphology [8]. Moreover, Ag can alter the function of mitochondria, changing the permeability of the phosphoryl membrane, and causing dysfunction of homeostasis, which results in the metabolic failure of the bacteria [9].

Generally, Ag nanoparticles are fabricated using various methods, including chemical, photo-thermal, electrochemical, and biological routes. The shape and size of Ag also influence its bactericidal activity. Numerous studies focused on how the size of Ag particles may affect their bactericidal efficiency [10,11]. At the same concentration, smaller Ag particles increase surface area and exhibit stronger bactericidal activity. Aside from the size factor, the shape and crystal structure crucially affect the surface chemistry [12,13]. For example, Dong et al. reported that the triangular Ag particles exhibited stronger bactericidal activity as compared with rod-shaped and spherical Ag [14]. They noted that the spherical Ag with (100) facets was less reactive than the triangular Ag with (111) facets.

Recently, we reported that Ag nanoparticles anchored on GO sheets can enhance bactericidal efficiency [8,15,16]. To date, in-depth antibacterial analyses of Ag NPrsms incorporated with GO have not been performed. In this study, Ag nanospheres and nanoprisms were prepared and grafted onto GO sheets. The physicochemical characteristics of the resulting GO–Ag composites were examined. The antibacterial properties of the pure GO, Ag particles and GO–Ag composites were evaluated via bacterial growth curves and a live/dead cell analysis on fluorescence microscopy. To further investigate the bacteria with and without nanomaterial treatment, bacterial morphology was evaluated using transmission electron microscopy (TEM) and field-emission scanning electron microscopy (FESEM). This study provides valuable information on how the shapes of Ag nanoparticles affect their antibacterial activity, and helps researchers in designing potent antibacterial materials.

## 2. Materials and Methods

### 2.1. Materials

Graphite powder, potassium permanganate (KMnO_4_), potassium bromide (KBr), hydrochloric acid (HCl), agar–agar powder, chitosan (CS), acetic acid (CH_3_COOH), glutaraldehyde solution 50% (GA), Hoechst (HS), propidium iodide (PI), dimethyl sulfoxide (DMSO), osmic acid (Os) 1%, phosphate-buffered saline (PBS), trisodium citrate (Na_3_C_6_H_5_O_7_), ethyl alcohol (C_2_H_5_OH), Luria–Bertani (LB) broth, concentrated sulfuric acid (H_2_SO_4_, 95–98%), and 2,7-dichlorofluorescein diacetate (DCFH_2_-DA) solution were purchased from Sigma-Aldrich, St. Louis, MO, USA. Silver nitrate (AgNO_3_) was purchased from Petal Plating Products and Labs Chemical, Mumbai, India. *Staphylococcus aureus* (*S. aureus*, BCRC 10781) and *Escherichia coli* (*E. coli*, DH5α) were purchased from the Bioresource Collection and Research Center (BCRC), Hsinchu, Taiwan.

### 2.2. Experimental Procedures

#### 2.2.1. GO Synthesis

GO was synthesized via Hummer’s method with slight modification [17]. Briefly, 2 g graphite powder was gradually dissolved in H_2_SO_4_ solution. The same amount of KMnO_4_ was sequentially added six times until the presence of a bright yellow-brown color in the mixture. Ice was added into the solution to reduce the reaction temperature. The precipitate settled at the bottom after several days, and the supernatant solution was removed. The precipitate was washed with HCl and deionized (DI) water alternately, and centrifuged until the solution became neutral pH. The product was collected and dried at 60 °C in a vacuum oven overnight.

#### 2.2.2. Synthesis of Silver Nanoparticles and Silver Nanoprisms

The silver nanoparticles (Ag NPs) were prepared using the previous procedure with slight modification [18]. First, an aliquot of 10 mL of 0.75 mM AgNO_3_ was mixed into 4 mL of 25 mM trisodium citrate solution. Subsequently, 5 mL of 10 mM NaBH_4_ was added. Then, 45 µL of 1 mM KBr was added to the mixture. Finally, the yellow color in the solution indicated that the Ag NPs were successfully prepared.

The silver nanoprisms (Ag NPrsms) were prepared according to the previous study with slight modification [19]. An aliquot of 10 mL of 0.75 mM AgNO_3_ was added into 4 mL of 25 mM of trisodium citrate solution, followed by the addition of 5 mL of 10 mM NaBH_4_. Subsequently, 5 mL of 0.05 mM H_2_O_2_ was added. The solution became blue in color, indicating the formation of Ag NPrsms.

#### 2.2.3. Synthesis of Silver Nanoparticle- and Silver Nanoprism-Loaded GO Nanosheets

The GO–Ag NPs were synthesized as follows. A total of 0.5 g GO was sonicated into 250 mL of deionized water for 30 min to form a homogenous solution. Thereafter, 100 mL of GO suspension was mixed with 25 mL of Ag NP solution in the presence of 4 mL CS (1 wt.%) solution to form the GO–Ag NPs. The solution was stirred for 6 h, then dialyzed using a dialysis tube to eliminate the unreacted Ag. The final product was washed many times with DI water, centrifuged and dried at 60 °C overnight in a vacuum oven. For the preparation of GO–Ag NPrsms, a similar process was employed except that the Ag NPs was replaced by Ag NPrsms.

### 2.3. Nanomaterial Characterizations

A UV-visible spectrophotometer (UV-vis, model V-650, JASCO Inc., Tokyo, Japan) was used to determine the absorption of the nanomaterial solutions. Their zeta potentials and particle size distributions were measured using a dynamic laser scattering analyzer (Zetasizer, model 2000 HAS, Malvern, Worcestershire, UK) in triplicate. Five microliters of 10 mM GO–Ag NPs and GO–Ag NPrsms were drop-cast on 200-mesh copper grids. A transmission electron microscope (TEM, model JEM-1230, JEOL Ltd., Tokyo, Japan) and a field emission scanning electron microscope (FESEM, model JSM-7500F, JEOL Ltd., Tokyo, Japan) equipped with an energy dispersive spectrometer (EDS, model E-MAX, Horiba Ltd., Tokyo, Japan) were used to observe the morphology of the samples. For higher resolution images and to differentiate the crystal structure of Ag particles, high resolution transmission electron microscopy (HRTEM, model JEM-2100Plus, JEOL Ltd., Tokyo, Japan) was employed with an accelerating voltage of 200 kV.

A thermogravimetric analyzer (TGA, model Q-500, TA Instrument Inc., New Castle, DE, USA) was used to analyze the mass loss in response to the temperature. X-ray diffraction (XRD, model D5005D, Siemens AG, Munich, Germany) was used to analyze the crystal structure of the materials. Fourier transform infrared spectroscopy (FTIR, model FT-730, Horiba Ltd., Kyoto, Japan) was used to determine the chemical bonding of the samples. X-ray photoelectron spectroscopy (XPS, MT-500, Thermo Fisher Scientific Inc., Waltham, MA, USA) was used to determine the elemental composition and functional groups of the samples.

### 2.4. Antibacterial Tests

#### 2.4.1. Agar Disk Diffusion Assay

Two strains of bacteria (*S. aureus* and *E. coli*) were incubated overnight in LB broth at 37 °C for 12 h and centrifuged at 3000 rpm for 2 min using a centrifugator (model Hermel Z326K, Labortechnik, New Taipei City, Taiwan). The supernatant was eliminated, leaving the precipitate. The precipitate was resuspended in LB broth and then diluted to obtain a solution with an optical density (OD) of 0.1. Initially, 25 mL of agar and LB medium mixture were filled into a Petri dish. An aliquot of 50 µL bacterial suspension was spread on the surface of the agar dish. Wells of 9 mm in diameter were punched in the agar dish and filled with 50 μL of tested samples with concentrations of 25, 50 and 100 μL mL^−1^. Each Petri dish was kept at 37 °C without shaking for 6 h. The inhibition zone was determined by measuring the diameter of the clear zone around the well.

#### 2.4.2. Optical Density (OD 600 nm) Measurement

Ten microliters of the aforementioned bacterial suspensions were diluted in 80 µL of LB broth. Another 10 µL of tested samples were added to the bacterial suspension and placed in a shaking incubator for 1 h and then transferred to 96-well plates. The OD results at time intervals were recorded using a microplate reader (Epoch, BioTek Instruments, Winooski, VT, USA) at a wavelength of 600 nm. The differences in OD values of the treated and untreated samples were analyzed in triplicates. The inhibition percentage was determined using the equation described in the literature [8].

#### 2.4.3. Morphological Changes in the Bacteria after Nanomaterial Exposure

The change in bacterial morphology upon nanomaterial exposure was investigated using TEM and FESEM analyses. The bacterial suspensions were incubated in nanomaterial solutions (50 μg mL^−1^) at 37 °C for 2 h, along with a copper grid or glass slide. Bacteria in LB solution were used as a control. Then, the bacteria-loaded copper grid or glass slide was transferred into a 12-well plate. The samples were fixed with 3% GA at 4 °C for 1 h. The samples were washed twice with PBS before being treated with 1% Os acid for another 45 min. The samples were then washed twice with PBS before dehydration using ethyl alcohol with an increasing concentration gradient of 25%, 50%, 75%, and 100% for 15 min each time. Critical point drying was performed on the samples prior to microscopy examination.

#### 2.4.4. Live/Dead Cell Analysis

To evaluate the population of bacteria, confocal laser scanning microscopy was used. The bacterial suspensions were incubated in nanomaterial solutions (50 μg mL^−1^) at 37 °C for 2 h. HS and PI dyes at a ratio of 10:1 (HS: PI) were mixed into the bacterial suspensions, which were then kept in the dark for 15 min. Following this procedure, the mixture was washed with PBS to remove residual dyes. The live/dead bacteria were observed under a confocal laser scanning microscope (Zeiss LSM 510-Meta, Heidelberg, Germany).

#### 2.4.5. Evaluation of Reactive Oxygen Species (ROS)

DCFH_2_-DA assay was utilized to determine the ROS production as described in our previous work [8]. The bacterial suspensions were incubated in nanomaterial solutions (50 μg mL^−1^) at 37 °C for 2 h and then were exposed to 5 µM DCFH_2_-DA in the dark. The mixture was incubated at 37 °C for 1 h, and the fluorescence images were examined using inverted microscopy (Eclipse TS 100 Inverted Routine Microscope, Nikon Instruments, London, UK).

## 3. Results and Discussion

### 3.1. Formulation of Ag Particles with Different Shapes

In the presence of NaBH_4_, Ag^+^ was reduced to Ag° according to the following reaction:

NaBH_4_ + 8AgNO_3_ + 4H_2_O → NaB(OH) _4_ + 8Ag° + 8HNO_3_

**Figure 1:**
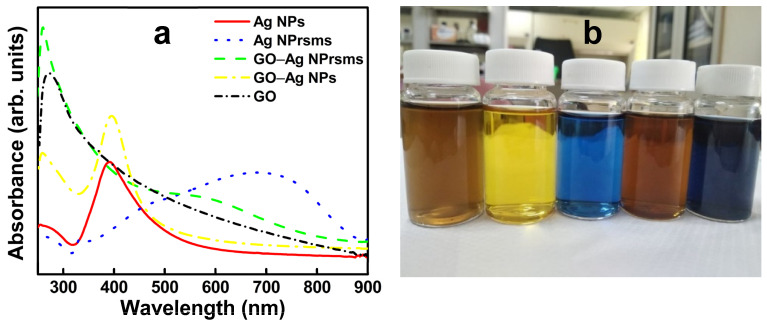
UV-vis spectra (**a**) and photo of nanomaterial suspensions (**b**) of GO, Ag NPs, Ag NPrsms, GO–Ag NPs, and GO–Ag NPrsms (from left to right).

The addition of trisodium citrate stabilized the Ag particles in the aqueous solution for citrate as a buffer and charge stabilizer [20]. In the preparation of Ag NPs, KBr was used to terminate the reaction and nuclei growth, and resulted in spherical shapes [21]. Successful Ag NPs exhibited a bright yellow color and light absorption at 400 nm (Figure 1). H_2_O_2_ is known as an etching agent [22,23] to favor prism shape formation by selectively dissolving unstable facets and yielding “seeds with planar twinned defects of stacking faults parallel to the (111) direction” [22], and the resulting solution of Ag NPrsms was blue in color with visible light absorption at 720 nm (Figure 1).

**Figure 2:**
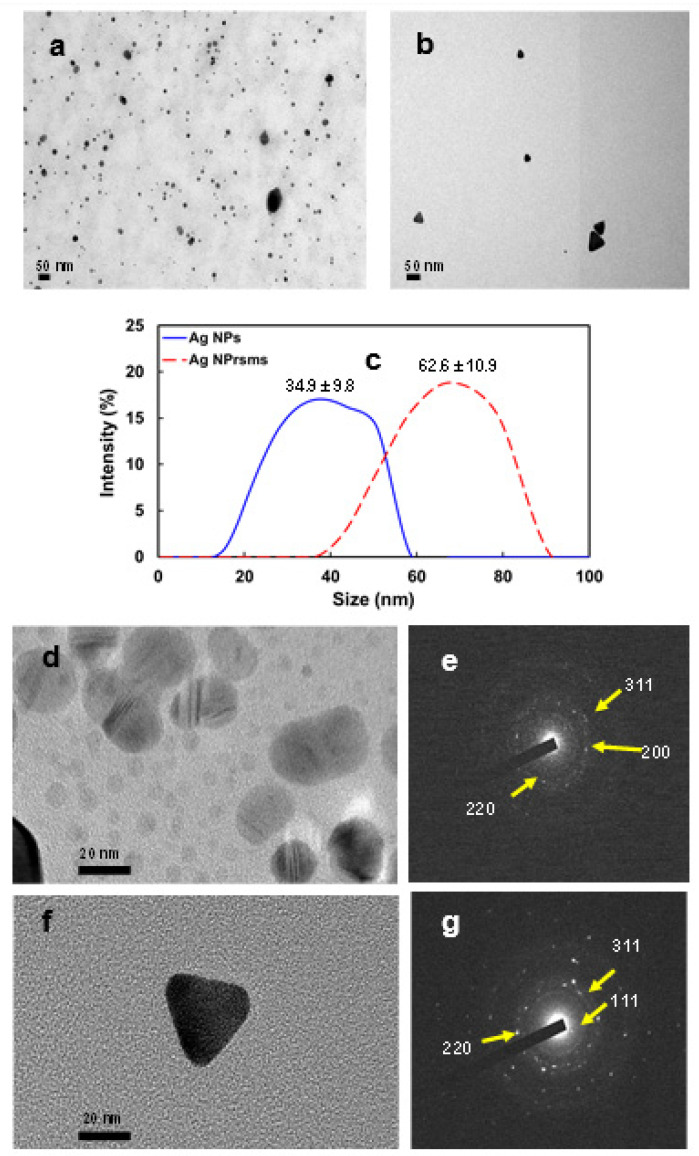
TEM images of Ag NPs (**a**) and Ag NPrsms (**b**); size distribution of Ag NPs and Ag NPrsms as measured using DLS (**c**); HRTEM of Ag NPs (**d**), SAED lattice pattern diffraction of Ag NPs (**e**), HRTEM of Ag NPrsms (**f**) and SAED lattice pattern diffraction of Ag NPrsms (**g**).

In the TEM images in Figure 2, the Ag NPs were of spherical shape (Figure 2a,d) and Ag NPrsms of sharp triangular shape (Figure 2b,f). The size of the Ag NPs was 20–40 nm, which was a good size for Ag NPs in biomedical and thermal therapy applications [13,24,25]. The Ag NPrsms appeared to be larger, at approximately 60 nm (TEM images in Figure 2b). The DLS analysis indicated that both Ag particles had similar size distributions but different mean sizes (Figure 2c). Both Ag particles also demonstrated similar zeta potential values: −21.7 and −22.2 mV for Ag NPs and Ag NPrsms, respectively. The selected area electron diffraction (SAED) patterns in Figure 2e,g indicate the crystal structure of Ag NPs and Ag NPrsms. The Ag NPs exhibited (200), (220) and (311) lattice planes. The Ag NPrsms showed (111), (220) and (311) lattices of high crystallinity. The (111) lattice plane appeared significantly in the Ag NPrsms.

### 3.2. Characterizations of GO Sheets Loaded with Ag Particles

**Figure 3:**
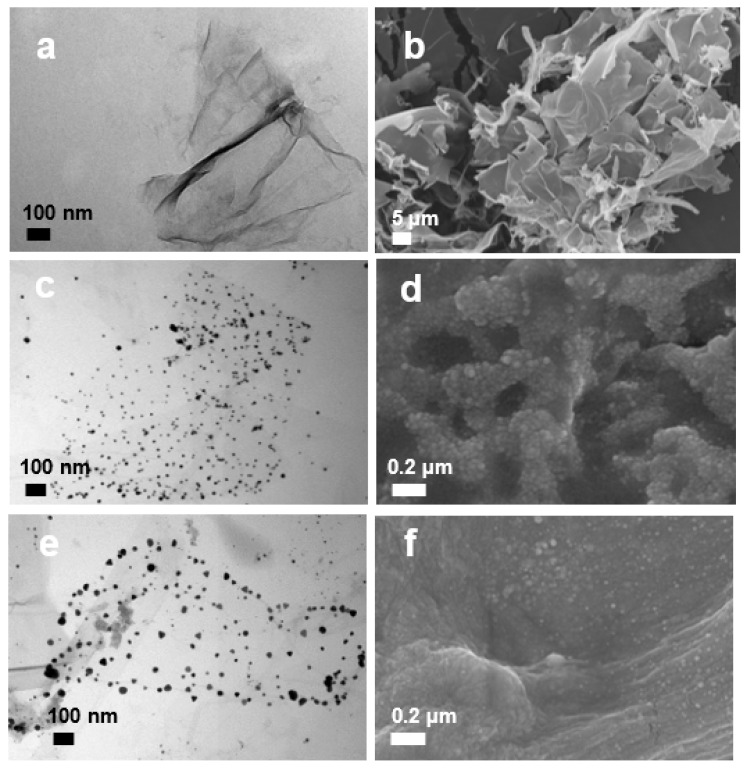
TEM and FESEM images of GO (**a**,**b**), GO–Ag NPs (**c**,**d**) and GO–Ag NPrsms (**e**,**f**), respectively.

The original GO solution was light brown in color, and its UV-visible spectrum showed a peak around 256 nm (Figure 1), which is typical due to the π-π* transition in sp^2^ structure. The as-prepared GO–Ag NP solution showed brown color, and its spectrum showed two peaks. One absorption peak around 256 nm derived from GO, and the other at 405 nm from Ag NPs. These absorption wavelengths of Ag NPs and GO–Ag NPs are in the range of those of Ag composites prepared via in situ reduction method [24]. The GO–Ag NPrsms demonstrated a dark blue color and had two absorption peaks: one from GO at 250 nm, and the other shoulder at 600–700 nm. The functional groups in GO may preferentially bind to (111) facets and prevent lattice growth, resulting in a truncated triangle shape. Such morphological and chemical bonding shifts may be associated with the blue shift of the Ag NPrsms in Figure 1a. Further observation using FESEM revealed the microstructure of the nanomaterials. As indicated in Figure 3a,b, the GO sheets were paper-like and typically wrinkled with single-layer structure. The Ag NPs and Ag NPrsms were anchored on GO and evenly distributed on the GO sheets (Figure 3c–f). Figures S1 and S2 clearly demonstrate the Ag distribution in GO–Ag NPs and GO–Ag NPrsms, respectively. In addition, EDS was employed to analyze the Ag contents. The Ag accounted for 29.1% (*w*/*w*) in the GO–Ag NPs and 23.7% in the GO–Ag NPrsms (Table S1).

**Figure 4:**
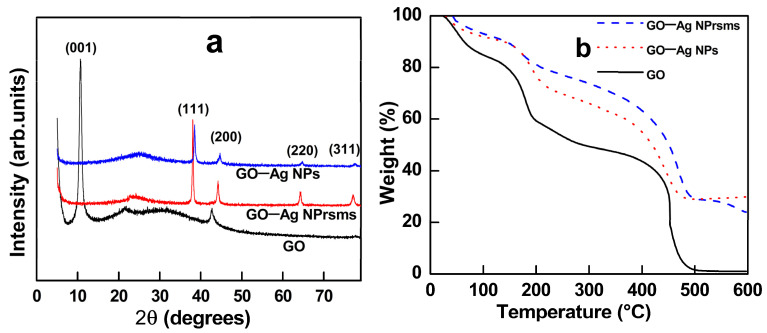
XRD profiles (**a**) and TGA thermograms (**b**) of GO, GO–Ag NPs, and GO–Ag NPrsms.

As shown in the XRD profiles in Figure 4a, GO–Ag NPs and GO–Ag NPrsms exhibited diffraction peaks at 38.1°, 44.4°, 64.6°, and 77.6°, representing the (111), (200), (220), and (311) crystal lattice structures, respectively. Kumar et al. used in-situ reduction of Ag^+^ by mixing AgNO_3_ precursor solution with modified halloysite nanotubes to form temperature-responsive nanomaterials [26]. In the current study, we used a chemical method to decorate GO with prepared Ag nanoparticles. Despite the different approaches to synthesis and preparation, their XRD spectra were in line with Figure 4a in this work.

A TGA analysis was used to investigate the materials’ thermal degradation behavior (Figure 4b). The GO showed three stages of mass losses. The first stage was from 30 to 100 °C, corresponding to the evaporation of water. The mass loss at approximately 180 °C was assigned to the decomposition of the oxygen-containing functional groups. The final mass loss at 420 °C was assigned to the loss of the remaining carbon from the graphene sheets [27]. The GO nanosheets completely decomposed in the air atmosphere. The GO composite containing Ag particles showed degradation temperatures similar to those of the GO sample, except that the residual masses remained at 600 °C: 30% in GO–Ag NPs and 23% in GO–Ag NPrsms.

**Figure 5:**
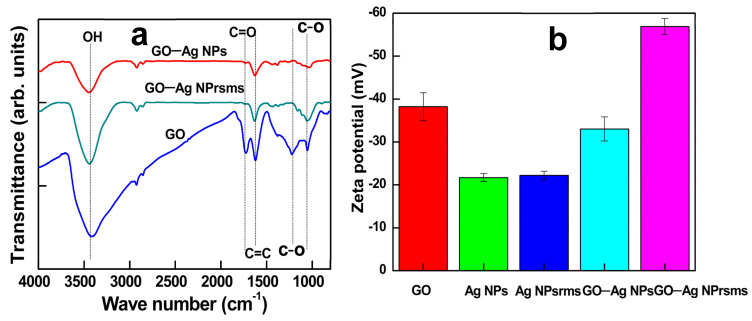
FTIR spectra of (**a**) of GO, GO–Ag NPs and GO–Ag NPrsms and zeta potential (**b**) of GO, Ag NPs, Ag NPrsms, GO–Ag NPs and GO–Ag NPrsms.

The FTIR spectrum of the GO revealed abundant oxygen-containing functional groups, and most of them were reduced or removed in the composites (Figure 5a). The peak at 1734 cm^−1^, which represents the C=O group, was removed, while the peaks at 1222 cm^−1^, corresponding to the C–O group, decreased in intensity. It is suspected that the carboxylic groups in GO may attract Ag cations for initial immobilization during composite formation.

**Figure 6:**
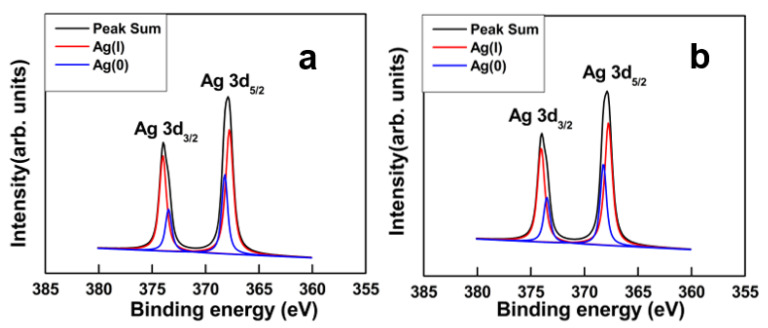
XPS spectra and deconvoluted peaks of Ag3d for GO–Ag NPs (**a**) and GO–Ag NPrsms (**b**).

The elemental compositions of the samples were examined using XPS analysis, as shown in the full scans in Figure S3a. The Ag peak appeared in both the GO–Ag NPs and GO–Ag NPrsms at mass percentages of 25.9% and 22.8%, respectively (Table S1). Figure 6a,b shows the high-resolution XPS spectra of the Ag 3d regions in the nanocomposites. The spectra show two peaks at 368.1 eV and 373.5 eV, which corresponded to Ag 3d_5/2_ and Ag 3d_3/2_, respectively [25,26]. The lower energy peak Ag 3d_5/2_ could be deconvoluted into two states: Ag (I) at 367.7 eV and Ag (0) at 368.5 eV. The 3d_3/2_ peak contained Ag (I) at 373.7 eV and Ag (0) at 373.2 eV. The deconvoluted peak areas were used to calculate their contributions. The GO–Ag NPrsms showed higher Ag (I) content (75.6%) than GO–Ag NPs (69.9%). The deconvolution of C1s peaks of GO, GO–Ag NPs and GO–Ag NPrsms composites are shown in Figure S3b–d, and related values in Table S2. It was clear that C-O and O–C–O groups were significantly reduced in the GO–Ag composites.

The zeta potential of GO was −38 mV. For GO–Ag NPs and GO–Ag NPrsms, the zeta potential values were −33 mV and −55 mV, respectively (Figure 5b). In the preparation of GO–Ag NPs, KBr was used, and the strong electrolyte may neutralize some negative charges, whereas in GO–Ag NPrsms synthesis, neutral H_2_O_2_ was employed, and the neutralization was limited, maintaining strong negative charges.

### 3.3. Antibacterial Test

#### 3.3.1. Agar Disk Diffusion Assay

**Figure 7:**
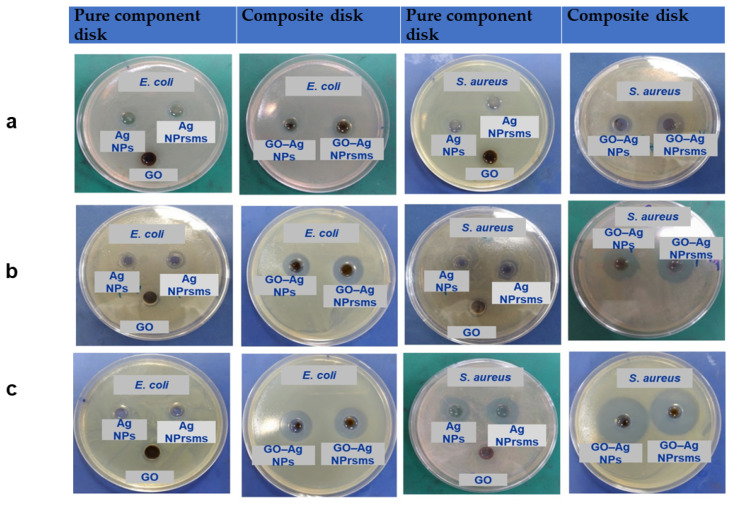
Inhibition zones of *E. coli* and *S. aureus* after treatment with GO, Ag NPs, Ag NPrsms, GO-Ag NPs, and GO-Ag NPrsms at nanomaterial concentrations of 25 µg mL^−1^ (**a**), 50 µg mL^−1^ (**b**), and 100 µg mL^−1^ (**c**).

Inhibition zones on the agar plates were determined for Gram-negative *E. coli* and Gram-positive *S. aureus* bacteria to evaluate the nanomaterials’ antibacterial activity. The inhibition zone diameters are indicated in Figure 7 and Table S3. For *E. coli* strain, the pure components (including GO, Ag NPs and Ag NPrsms) showed no inhibition zone at a low concentration of 25 µg mL^−1^. The inhibition zone was measured at 11 and 14 mm for GO–Ag NPs and GO–Ag NPrsms samples, respectively. At the concentration of 50 µg mL^−1^, both GO and Ag NPs did not show an inhibition zone, while Ag NPrsms exhibited an inhibition zone of 11 mm. The clearer and bigger zones at 13 and 13.5 mm were observed for GO–Ag NPs and GO–Ag NPrsms samples, respectively. At the high concentration of 100 µg mL^−1^, GO was found to exhibit a slight inhibition zone (10 mm), as well as Ag NPs (10.5 mm) and Ag NPrsms (12 mm). In contrast, the GO–Ag NPs and GO–Ag NPrsms demonstrated the best antibacterial effect with inhibition zones of 20 mm and 20.5 mm, respectively. 

The inhibition zones of *S. aureus* after treatment with nanomaterials are shown in Figure 7. The GO sample resulted in an inhibition diameter of 10, 11 and 12 mm at 25, 50 and 100 µg mL^−1^, respectively. As for Ag NPs, the inhibition zones were 11, 12 and 17.5 mm at the same doses. The Ag NPrsms exhibited larger zones of 11, 13 and 19.5 mm for 25, 50 and 100 µg mL^−1^. For the composite groups, the inhibition was more significant. Even at a small concentration of 25 µg mL^−1^, GO–Ag NPrsms exhibited a highly bactericidal effect (18 mm), higher than that of GO–Ag NPs (15 mm). As the concentration increased to 100 µg mL^−1^, both the composites demonstrated larger zones: 31 mm and 33 mm for GO–Ag NPs and GO–Ag NPrsms, respectively. Table S3 summarizes the diameters of the inhibition zones. Increasing nanomaterial concentrations enhanced the bactericidal efficiency, and the nanomaterial efficacy was in the following order: GO–Ag NPrsms > GO–Ag NPs > Ag NPrsms > Ag NPs > GO. In addition, *E. coli* were more resistant than *S. aureus*.

#### 3.3.2. OD 600 nm Measurement

**Figure 8:**
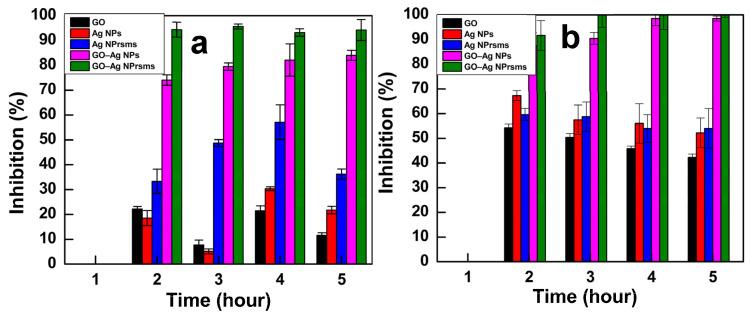
Inhibition values for *E.* *coli* (**a**) and *S. aureus* (**b**) after treatment with GO, Ag NPs, Ag NPrsms, GO-Ag NPs, and GO-Ag NPrsms nanomaterials at 100 µg mL^−1^ dose (*n* = 3).

A time-dependent antibacterial assay under an optical density (OD) at a 600 nm wavelength against *E. coli *and *S. aureus* was utilized to determine the inhibition percentage over time. Bacteria without nanomaterial treatments were used as a control. The entire bacterial colonies after treatment were reduced as compared with the control (as indicated in Figure S3). After 5 h exposure, the *E. coli *growth was inhibited by 11.2, 18.5, 35, 72.9 and 93.2% for GO, Ag NPs, Ag NPrsms, GO–Ag NPs and GO–Ag NPrsms, respectively. The *S. aureus *population was reduced by 42.6, 52, 54.4, 98.5 and 100% by the GO, Ag NPs, Ag NPrsms, GO–Ag NPs and GO–Ag NPrsms, respectively (Figure 8b). 

Herein, the antimicrobial inhibition exhibited a steadily rising trend for GO, Ag NPs, Ag NPrsms, and GO–Ag NPs and reached the highest level of inhibition in the GO–Ag NPrsms sample. Similarly to the results of the disk diffusion assay, GO–Ag NPrsms were the most efficient inhibitors, and *E. coli* was more resistant than *S. aureus*.

**Figure 9:**
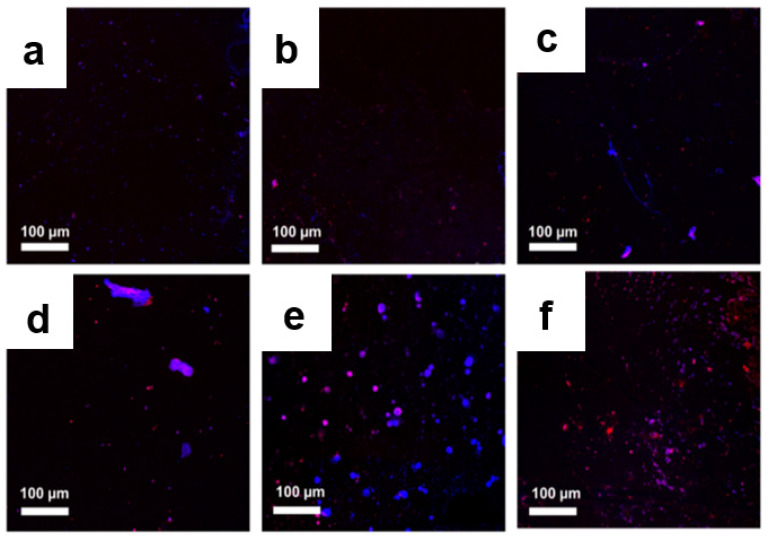
Confocal fluorescent images of control *E. coli* (**a**) and *E. coli* after treatment with GO (**b**), Ag NPs (**c**), Ag NPrsms (**d**), GO–Ag NPs (**e**), and GO–Ag NPrsms (**f**) stained with PI (red) and HS (blue).

**Figure 10:**
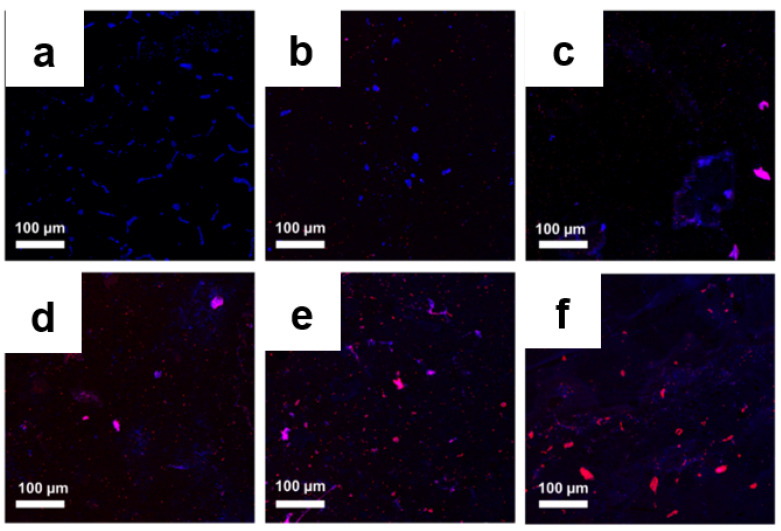
Confocal fluorescent images of control *S. aureus* (**a**) and *S. aureus* treated with GO (**b**), Ag NPs (**c**), Ag NPrsms (**d**), GO–Ag NPs (**e**), and GO–Ag NPrsms (**f**) stained with PI (red) and HS (blue).

To determine the quantity and quality of the bacterial colony before and after treatment, further microscopy observations were performed using a live/dead cell assay. Bacteria with an intact cell membrane were stained blue using HS, whereas damaged bacteria were stained red using PI. A large number of live bacteria were observed in the control group, as shown in Figure 9a and Figure 10a. In the GO-treated group, almost all bacteria were found to be alive, whereas a larger number of dead bacteria were found in Ag NPs and Ag NPrsms groups, which indicated a weak-to-moderate antibacterial performance of the pure components (Figure 9b–d and Figure 10b–d). In contrast, dead bacteria were found in the GO–Ag NPs and GO–Ag NPrsms groups (Figure 9e,f and Figure 10e,f). More specifically, treatment with GO–Ag NPrsms produced the highest bactericidal activity. Evidently, the ratio of dead cells was higher for *S. aureus* (Figure 10) than for *E. coli* (Figure 9). Figure 10f shows that most of the dead bacteria were *S. aureus* after incubating with GO–Ag NPrsms.

### 3.4. Bacterial Morphology Changes after Treatment with Nanomaterials

**Figure 11:**
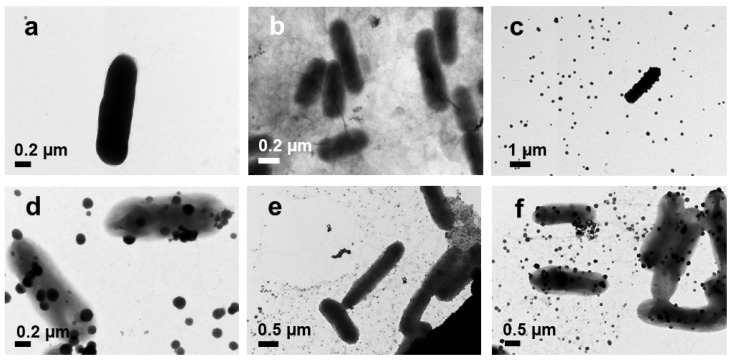
TEM images of *E. coli* before (**a**) and after treatment with GO (**b**), Ag NPs (**c**), Ag NPrsms (**d**), GO–Ag NPs (**e**), and GO–Ag NPrsms (**f**).

**Figure 12:**
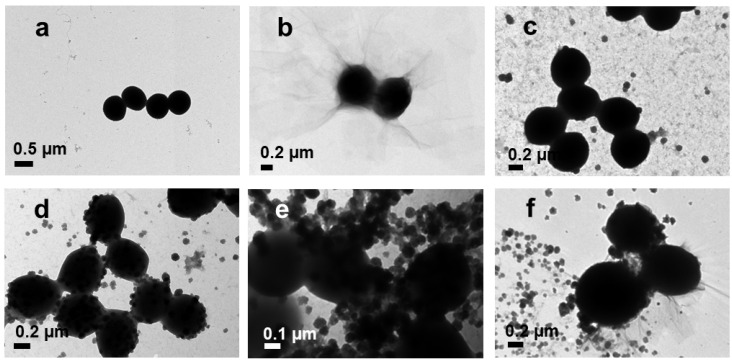
TEM images of *S. aureus* before (**a**) and after treatment with GO (**b**), Ag NPs (**c**), Ag NPrsms (**d**), GO–Ag NPs (**e**), and GO–Ag NPrsms (**f**).

TEM was used to observe the morphological changes in the bacteria before and after treatment with nanoparticles. The control *E. coli* had a typical rod shape (Figure 11a), and *S. aureus* had a spherical shape (Figure 12a). After treatment with GO, *E. coli* and *S. aureus* were attached with the GO layers (Figure 11b and Figure 12b). The Ag NPs and Ag NPrsms came into direct contact with the bacteria and accumulated on the outer membranes (Figure 11c and Figure 12c). The bacterial cells after treatment with GO–Ag NPs and GO–Ag NPrsms experienced severe deformation, losing their typical shape and structure, causing intracellular content to leach out.

**Figure 13:**
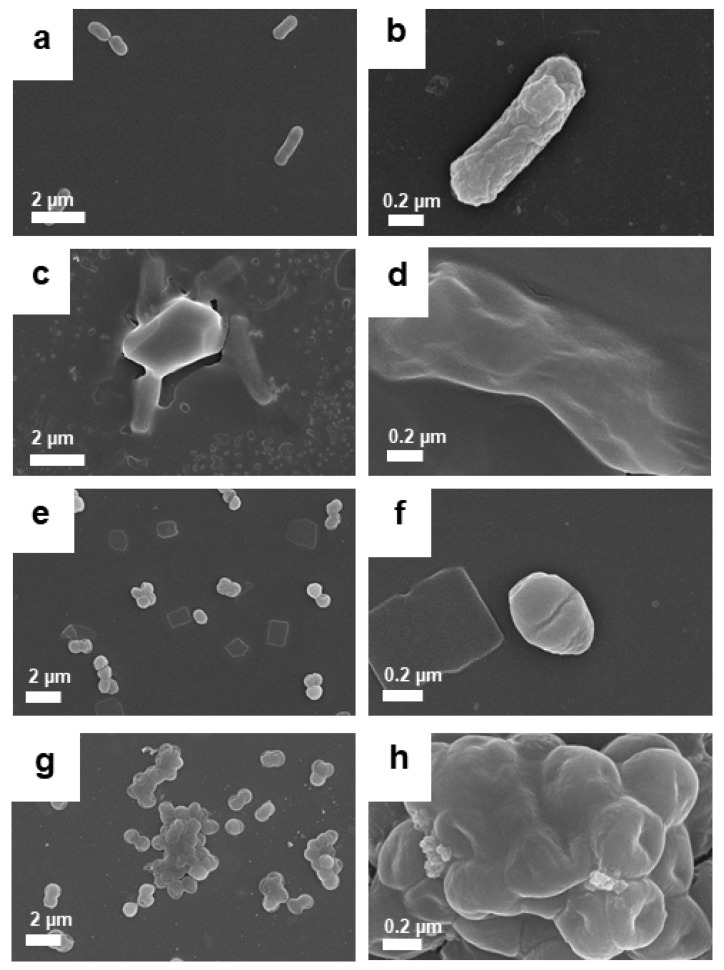
FESEM images of *E. coli* before (**a**,**b**) and after treatment (**c**,**d**) with GO–Ag NPrsms, and *S. aureus* (**e**,**f**) before and after treatment (**g**,**h**) with GO–Ag NPrsms.

A clearer observation was achieved using FESEM, as illustrated in Figure 13. Both *E. coli* and *S. aureus* lost their shape after treatment with GO–Ag NPrsms (Figure 13c,d,g,h) as compared with the control bacteria (Figure 13a,b,e,f). In detail, the *E. coli* appeared to have been cut into shorter, smaller pieces, and the cell membranes were damaged (Figure 13c,d). The *S. aureus* treated with GO–Ag NPrsms were found in cluster agglomerations, losing their classical spherical shape (Figure 13g,h).

In addition, this research showed that Gram-negative *E. coli* exhibited more resistance than *S. aureus*. The impermeable outer membrane of *E. coli* protects the cell wall peptidoglycan, limiting nanoparticle attachment and bactericidal activity. The lack of an outer membrane in *S. aureus* allows easier charge-transfer charge and makes it more vulnerable to nanomaterials [28,29]. Moreover, the ARN reflux of the cytoplasm in *S. aureus* comes into direct contact with the edge of the GO nanowalls, which causes more damage as compared with Gram-negative *E. coli* [30].

### 3.5. Reactive Oxygen Species Production

**Figure 14:**
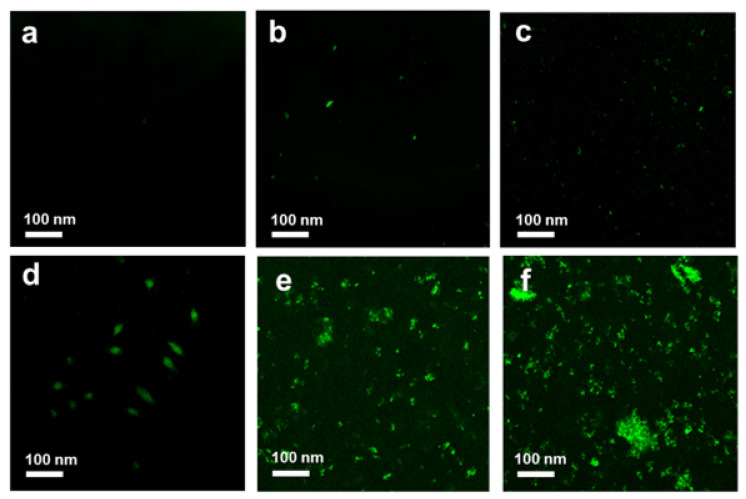
ROS fluorescence images of control *S. aureus* (**a**) and *S. aureus* treated with GO (**b**), Ag NPs (**c**), Ag NPrsms (**d**), GO–Ag NPs (**e**), and GO–Ag NPrsms (**f**) stained with DCFH-DA.

In order to explore the relationship between ROS and bactericidal activity, a DCFH-DA assay was conducted. ROS cause an imbalance between the production of reactive oxygen and the ability of the biological system to repair damage, leading to disorders in the functioning of the entire bacterial system [31]. The control *S. aureus* showed no ROS produced in culture, as indicated in Figure 14a. When the bacteria were exposed to GO, some ROS were produced, as shown in Figure 14b. More green dots were found in the Ag NPs treatment, indicating a higher amount of ROS for the Ag NPs group than the GO group (Figure 14c). The ROS level increased after bacterial exposure to Ag NPrsms. This ROS production from the Ag NPs was higher than that from GO, as was evidenced in previous work [8]. Significant ROS were induced by the composite groups, whereby greater green fluorescence was noted as compared with that induced by the pure Ag or GO groups (Figure 14e,f). The highest ROS assembly was achieved in the GO–Ag NPrsms sample (Figure 14f). This result also suggests that Ag NPs, Ag NPrsms, GO–Ag NPs, and GO–Ag NPrsms can mediate the oxidation of DCFH-DA at different levels, which is in accordance with the antibacterial results.

### 3.6. Comparison of Ag Particles of Different Shapes and Their GO Composites

Many research works have demonstrated that Ag NPrsms have higher bactericidal effects than Ag NPs [14,32,33,34] due to the high densities of their (111) lattice planes [32,33]. Pham et al. prepared Ag NPs and Ag NPrsms and reported their MIC values of 6 and 4 µg mL^−1^, respectively [14]. Other authors reported that Ag NPrsms exhibited antibacterial efficiency via a photothermal ability [35,36]. Gold nanoprisms were reported to have good bactericidal activities and biofilm prevention because they possess plasmonic properties [37]. Recently, Singh et al. demonstrated the potency of ZnO triangular nanoparticles in combating harmful pathogens [38]. These ZnO nanoprisms may produce high quantities of ∙OH and ∙O^2−^ free radicals, resulting in antibacterial activity [39]. Generally, Ag with a smaller size and spherical shape has a higher surface energy than triangular Ag. Higher surface energies are unstable, so Ag NPs easily agglomerate [40] and diffuse into bacteria at a slow rate [41], thus reducing their antibacterial effect when compared with Ag NPrsms. The above explanations are possible mechanisms for Ag NPrsms’ superior bactericidal performance. We reported for the first time that Ag NPrsms produced higher ROS than Ag NPs.

Both *S. aureus* and *E. coli* after treatment with GO–Ag composites were destroyed more severely than those treated with pure GO or Ag (Figure 11 and Figure 12). Similar synergistic antibacterial function was observed with the GO–Ag NPrsms, as we reported earlier for GO–Ag NPs [8]. This booster antibacterial effect was due to the combination of bacteriostatic Ag particles and GO as the scaffold for Ag attachment. *S. aureus* treated with GO–Ag composites experienced severe damage, with stronger green fluorescent intensities than those achieved by treatment with the pure components. The antibacterial efficiency was parallel to the ROS level. The high ROS promoted the oxidizing agent production for bactericidal activity, inhibiting the respiration process, disrupting DNA translation, and causing bacterial apoptosis [42].

The GO–Ag NPrsms showed antibacterial activity superior to that of GO–Ag NPs. The nanoprisms possessed high density of (111) basal plane (Figure 2g and Figure 4a), beneficial for bacterial inhibition. Furthermore, the Ag (I) content in GO–Ag NPrsms (75.6%) was higher than that in GO–Ag NPs (69.9%). According to Chook et al., the high Ag (I) in the composite could facilitate higher bactericidal effect than in Ag (0) form [43]. For GO–Ag NPrsms, the good antibacterial activity was due to the high Ag (I) content and high ROS production.

## 4. Conclusions

Spherical silver nanoparticles (Ag NPs) and silver nanoprisms (Ag NPrsms) were synthesized and decorated on graphene oxide (GO) nanosheets to form nanocomposites as bactericidal agents. The Ag contents were 29% and 23% in the GO–Ag NPs and GO–Ag NPrsms, respectively. The Ag crystalline structure, size and shape in the nanocomposites resembled those of the pristine Ag particles. The Ag NPrsms exhibited a stronger (111) crystal signal than the Ag NPs. The GO–Ag NPrsms exhibited higher Ag (I) content (75.6%) than the GO-Ag NPs (69.9%). Increasing the nanomaterial concentration from 25 to 100 µg mL^−1^ improved the bactericidal efficiency, and the antibacterial potency was in the following order: GO–Ag NPrsms > GO–Ag NPs > Ag NPrsms > Ag NPs > GO. Gram-positive *Staphylococcus aureus* (*S. aureus*) was more vulnerable than Gram-negative *Escherichia coli* (*E. coli*) upon exposure to these nanomaterials. The GO–Ag NPrsms demonstrated a complete (100%) bactericidal effect against *S. aureus* at a concentration of 100 µg mL^−1^. The bacteria treated with GO–Ag NPrsms exhibited flattened structures and a loss of original morphology. The GO–Ag composites outperformed Ag or GO due to the synergistic effect of bacteriostatic Ag particles and GO affinity toward bacteria. The levels of reactive oxygen species produced in the bacteria–nanomaterial mixtures were highly correlated to the antibacterial efficacy values. The GO–Ag NPrsms are promising as bactericidal agents to suppress biofilm formation and inhibit bacterial infection. The antibacterial composites demonstrated potential in biomedical and clinical applications.

## Data Availability

Not applicable.

## Supplementary Materials

The following supporting information can be downloaded at: https://www.mdpi.com/article/10.3390/pharmaceutics14050924/s1, Figure S1: FESEM image and Ag mapping in GO–Ag NPs and elemental composition therein; Figure S2: FESEM image and Ag mapping in GO–Ag NPrsms and elemental composition therein; Figure S3: XPS (d); full scans of GO, GO–Ag NPs and GO-Ag NPrsms (a); Detailed C1s scans and deconvoluted peaks of GO (b), GO–Ag NPs (c), and GO–Ag NPrsms Figure S4: Time-dependent OD values of *E. coli* (a) and S. aureus (b) treated with nanomaterials at concentration of 100 µg mL^−1^; Table S1: Ag contents (% *w/w*) in GO–Ag NPs and GO–Ag NPrsms by TGA, EDS and XPS analyses; Table S2: Carbon functional group contribution in GO, GO–Ag NPs, and GO–Ag NPrsms, determined from XPS C1s peak deconvolution (Figure S3); Table S3: Inhibition zone diameters on agar plates after *E. coli* and *S. aureus* exposure with nanomaterial samples.
