# Supplementary material for: Prismatic Silver Nanoparticles Decorated on Graphene Oxide Sheets for Superior Antibacterial Activity
Source: Pharmaceutics. 2022 Apr 24;14(5):924. doi: 10.3390/pharmaceutics14050924 (PMC9147939; doi:10.3390/pharmaceutics14050924)
Supplement: Supplementary file 1 [file pharmaceutics-14-00924-s001.zip › pharmaceutics-1614099-supplementary.pdf]

SUPPORTING INFORMATION

Prismatic Silver Nanoparticles Decorated on Graphene Oxide Sheets for Superior Antibacterial Activity

Thi Tuong Vi Truong [1,2], Chien-Chang Chen [1,3#], Selvaraj Rajesh Kumar[2], Chih-Chien Hu [4], Dave W. Chen [5], Yu-Kuo Liu [2] and Shingjiang Jessie Lue [2,5,6], *

[1] Division of Pediatric Gastroenterology and Hepatology, Department of Pediatrics, Chang Gung Memorial Hospital, Taoyuan 333, Taiwan; truongthituongvi005@gmail.com (T.T.V.T.); cgj2841@cgmh.org.tw (C.-C.C.)
[2] Department of Chemical and Materials Engineering, Chang Gung University, Taoyuan City 333, Taiwan; rajeshkumarnst@gmail.com (S.R.K); ykliu@mail.cgu.edu.tw (Y.-K.L.)
[3] College of Medicine, Chang Gung University, Taoyuan 333, Taiwan;
[4] Division of Joint Reconstruction, Department of Orthopedics, Chang Gung Memorial Hospital, Taoyuan 333, Taiwan; chihchienhu@hotmail.com (C.-C.H)
[5] Department of Orthopedic Surgery, Chang Gung Memorial Hospital, Keelung City 204, Taiwan; mr5181@cgmh.org.tw (D.W.C)
[6] Department of Safety, Health and Environment Engineering, Ming-Chi University of Technology, New Taipei City 243, Taiwan
* Correspondence: jessie@mail.cgu.edu.tw (S.J.L); Tel.: +866-3-211-8800 (ext. 5489); Fax: +886-3-211-8700

# Contributed equally to first author

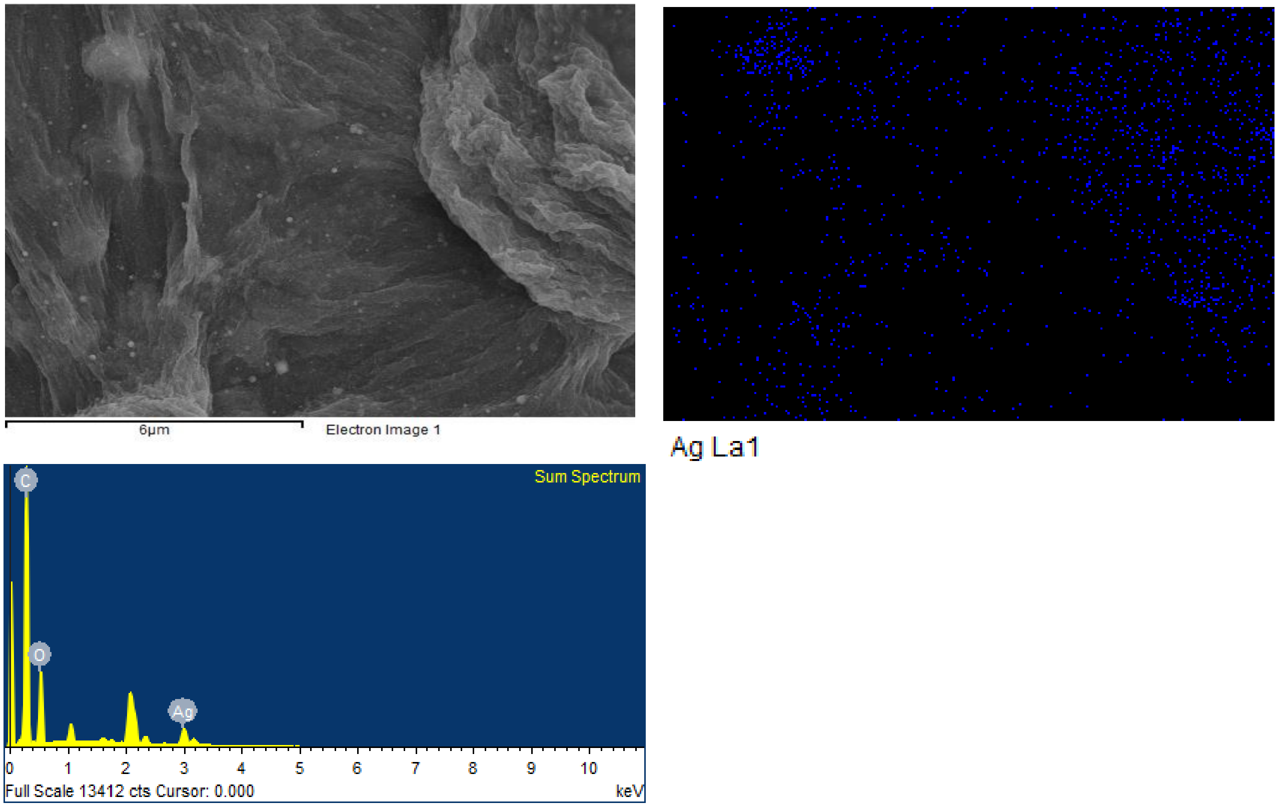

Figure S1. FESEM image and Ag mapping in GO–Ag NPs and elemental composition therein.

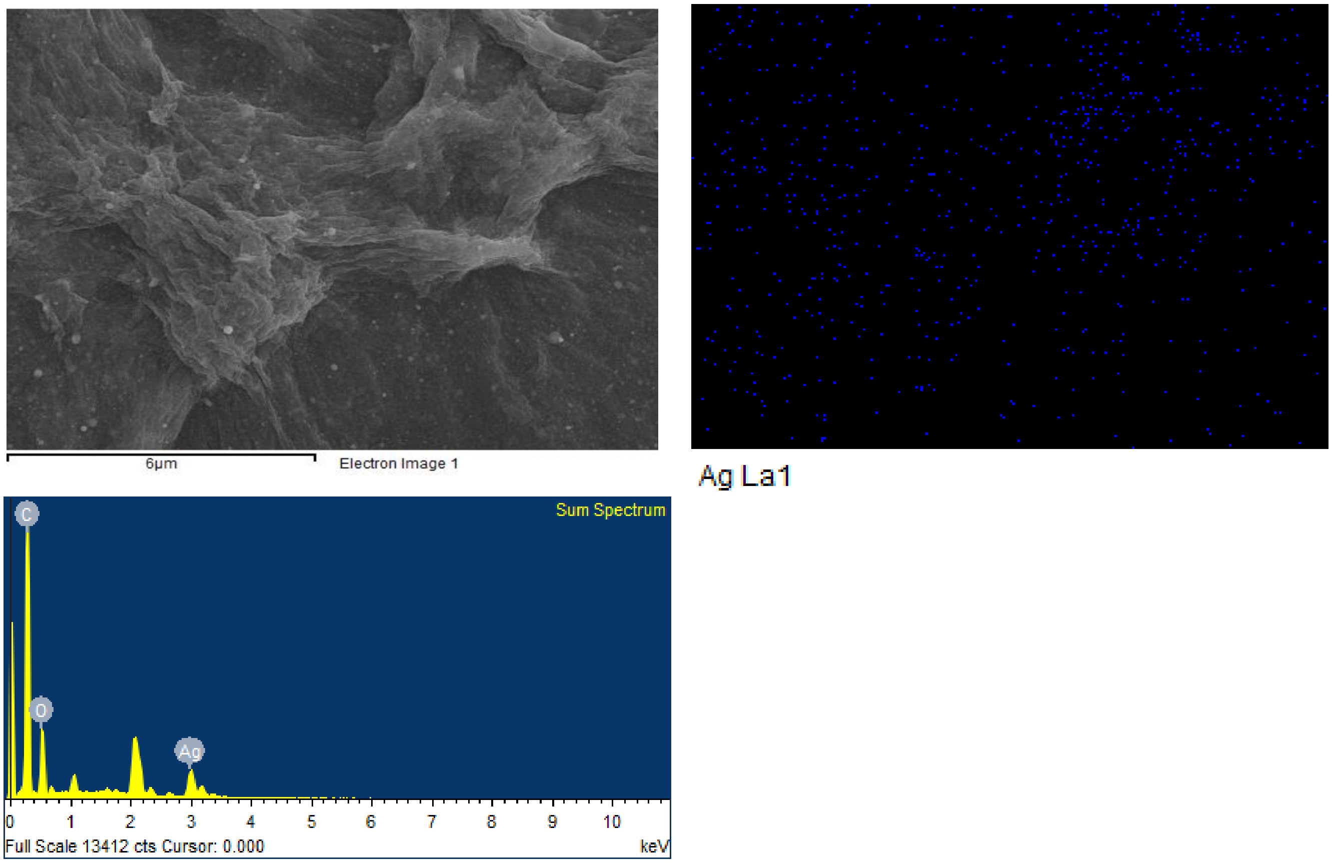

Figure S2. FESEM image and Ag mapping in GO–Ag NPrsms and elemental composition therein.

Table S1. Ag contents (% w/w) in GO–Ag NPs and GO–Ag NPrsms by TGA, EDS and XPS analyses.

Sample	TGA	EDS	XPS
**Ag NPs**	29.6	29.1	25.9
**Ag NPrsms**	23.3	23.7	22.8

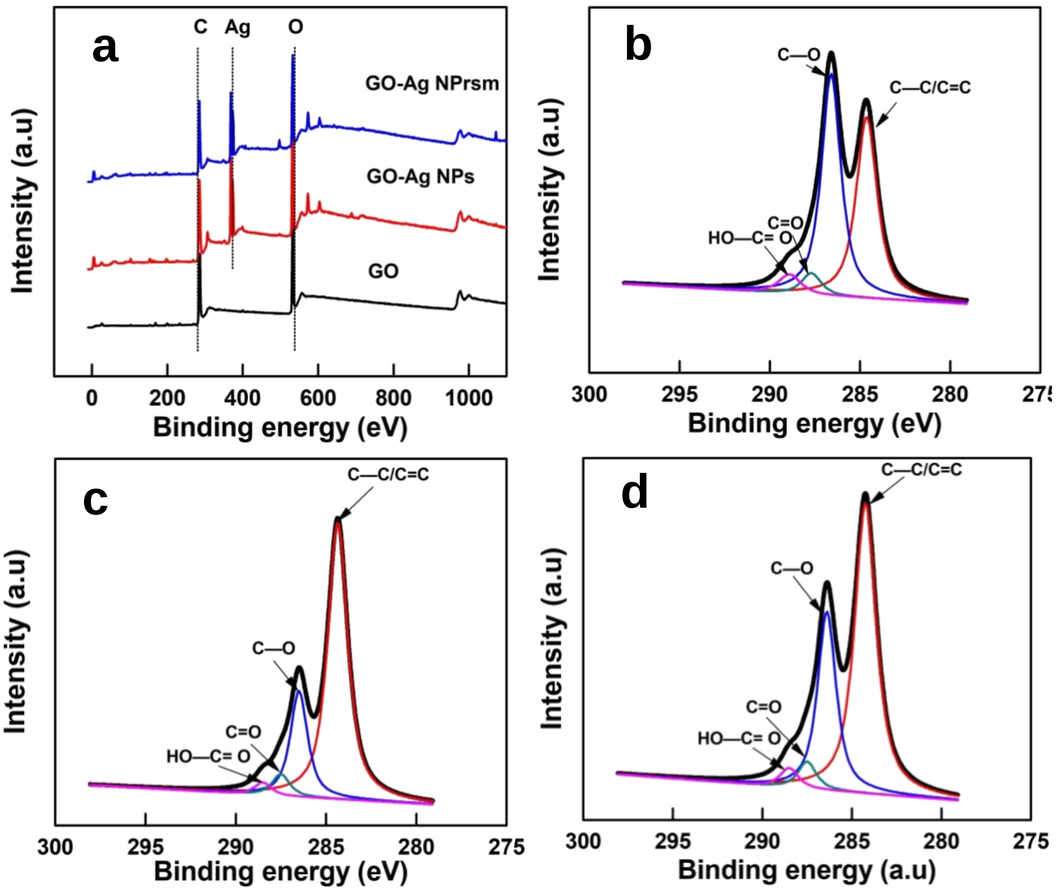

Figure S3. XPS full scans of GO, GO-Ag NPs and GO-Ag NPrsms (a); Detailed C1s scans and deconvoluted peaks of GO (b), GO-Ag NPs (c), and GO–Ag NPrsms (d).

Table S2. Carbon functional group contribution in GO, GO–Ag NPs, and GO–Ag NPrsms, determined from XPS C1s peak deconvolution (Figure S3).

C bonding	C=C	C–O	C=O	O–C–O
**GO**	42.3	**48.1**	4.8	4.8
**GO–Ag NPs**	69.6	23.0	**4.8**	**2.6**
**GO–Ag NPrsms**	60.3	**31.5**	**4.7**	**3.5**

Table S3. Inhibition zone diameters on agar plates after *E. coli* and *S. aureus* exposure with nanomaterial samples.

**Bacteria**	**Concentration**	**GO**	**Ag NPs**	**Ag NPrsms**	**GO–Ag NPs**	**GO–Ag NPrsms**
E. coli	$25\ \mu g\ mL^{-1}$	_ [a]	_ [a]	_ [a]	11.0	14.0
	$50\ \mu g\ mL^{-1}$	_ [a]	_ [a]	11.0	13.0	13.5
	$100\ \mu g\ mL^{-1}$	10.0	10.5	12	20	20.5
S. aureus	$25\ \mu g\ mL^{-1}$	10.0	11.0	11.0	15.0	18.0
	$50\ \mu g\ mL^{-1}$	11.0	12.0	13.0	22.0	23.0
	$100\ \mu g\ mL^{-1}$	12.0	17.5	19.5	31.0	33.0

[a] _ No inhibition zone was observed.

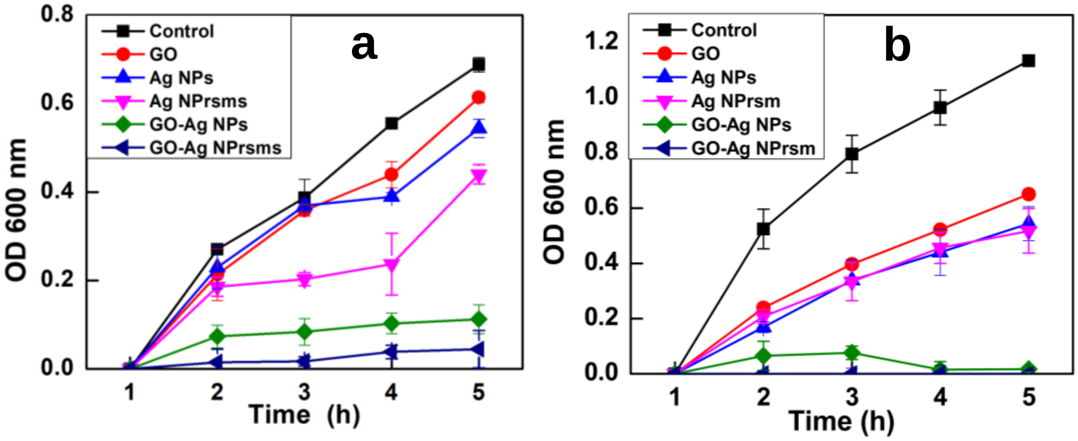

Figure S4. Time-dependent OD values of *E. coli* (a) and *S. aureus* (b) treated with nanomaterials at concentration of 100 μg mL^{-1}.
